# The Clinical Efficacy and Safety of Tulsi in Humans: A Systematic Review of the Literature

**DOI:** 10.1155/2017/9217567

**Published:** 2017-03-16

**Authors:** Negar Jamshidi, Marc M. Cohen

**Affiliations:** School of Health and Biomedical Sciences, RMIT University, Melbourne, VIC, Australia

## Abstract

Tulsi, also known as holy basil, is indigenous to the Indian continent and highly revered for its medicinal uses within the Ayurvedic and Siddha medical systems. Many in vitro, animal and human studies attest to tulsi having multiple therapeutic actions including adaptogenic, antimicrobial, anti-inflammatory, cardioprotective, and immunomodulatory effects, yet to date there are no systematic reviews of human research on tulsi's clinical efficacy and safety. We conducted a comprehensive literature review of human studies that reported on a clinical outcome after ingestion of tulsi. We searched for studies published in books, theses, conference proceedings, and electronic databases including Cochrane Library, Google Scholar, Embase, Medline, PubMed, Science Direct, and Indian Medical databases. A total of 24 studies were identified that reported therapeutic effects on metabolic disorders, cardiovascular disease, immunity, and neurocognition. All studies reported favourable clinical outcomes with no studies reporting any significant adverse events. The reviewed studies reinforce traditional uses and suggest tulsi is an effective treatment for lifestyle-related chronic diseases including diabetes, metabolic syndrome, and psychological stress. Further studies are required to explore mechanisms of action, clarify the dosage and dose form, and determine the populations most likely to benefit from tulsi's therapeutic effects.

## 1. Introduction

Tulsi in Hindi or Tulasi in Sanskrit (holy basil in English) is a highly revered culinary and medicinal aromatic herb from the family Lamiaceae that is indigenous to the Indian subcontinent and been used within Ayurvedic medicine more than 3000 years. In the Ayurveda system tulsi is often referred to as an “Elixir of Life” for its healing powers and has been known to treat many different common health conditions. In the Indian Materia Medica* tulsi* leaf extracts are described for treatment of bronchitis, rheumatism, and pyrexia [1]. Other reported therapeutic uses include treatment of epilepsy, asthma or dyspnea, hiccups, cough, skin and haematological diseases, parasitic infections, neuralgia, headache, wounds, and inflammation [2] and oral conditions [3]. The juice of the leaves has been applied as a drop for earache [4], while the tea infusion has been used for treatment of gastric and hepatic disorders [5]. The roots and stems were also traditionally used to treat mosquito and snake bites and for malaria [5].

Three types of tulsi are commonly described.* Ocimum tenuiflorum* (or* Ocimum sanctum* L.) includes 2 botanically and phytochemically distinct cultivars that include Rama or Sri tulsi (green leaves) and Krishna or Shyama tulsi (purplish leaves) [6, 7], while* Ocimum gratissimum* is a third type of tulsi known as Vana or wild/forest tulsi (dark green leaves) [8, 9]. The different tulsi types exhibit vast diversity in morphology and phytochemical composition including secondary metabolites, yet they can be distinguished from other* Ocimum* species by the colour of their yellow pollen, high levels of eugenol [10], and smaller chromosome number [11]. Despite being distinct species with* Ocimum tenuiflorum* having six times less DNA than* Ocimum gratissimum* [11], they are traditionally used in the same way to treat similar ailments [5]. For consistency, this review uses the term tulsi to refer to both* Ocimum tenuiflorum* or* Ocimum gratissimum*.

Tulsi has been the subject of numerous scientific studies and its pharmacological and wide range of therapeutic applications are the subject of more than one hundred publications during the last decade alone. Numerous in vitro and animal studies attest to tulsi leaf having potent pharmacological actions that include adaptogenic [12–14], metabolic [15–17], immunomodulatory [18–20], anticancer [21–23], anti-inflammatory [24, 25], antioxidant [26, 27], hepatoprotective [28, 29], radioprotective [30, 31], antimicrobial [32–35], and antidiabetic effects [36–38] that have been extensively reviewed previously [39–45].

Preclinical studies have demonstrated that tulsi increases swimming survival times in mice and prevents stress-induced ulcers in rats [46] with antistress effects comparable to antidepressant drugs [47]. Similarly, recent studies report leaf extracts from ethanolic and aqueous tulsi protects rats from stress-induced cardiovascular changes [48, 49]. Studies in animal models have further shown that the leaf extract of tulsi possesses anticonvulsant and anxiolytic activities [50, 51]. Several animal studies conducted over the past fifty years report that ingestion of tulsi leaves improves both glucose and lipid profiles in normal and diabetic-induced animal models [36, 38, 52–58]. Tulsi aqueous leaf extract intramammary infusion has also showed promising effect on improving the immune response in bovine models [59].

In addition to the extensive literature documenting in vitro and animal research, studies on the use of tulsi as part of a polyherbal formulation in humans has been systematically reviewed [60]. To date, however, there are no systematic reviews on the clinical efficacy and safety of tulsi as a single herbal intervention in humans. The objective of this review was therefore to summarize and critically appraise human clinical trials of tulsi in order to assess the current evidence on tulsi's clinical efficacy and safety.

## 2. Methods and Materials

### 2.1. Search Strategies

Relevant clinical studies were identified through searching PubMed, Google Scholar, ScienceDirect, Medline, Embase, Cochrane Library, and Indian Medical databases. The terms in the title or abstract (MeSH and free search terms) alone or in combination searched were “Tulsi”, “Tulasi”, “Holy Basil”, “ocimum sanctum”, “Ocimum tenuiflorum”, “Ocimum gratissimum”, “ocimum”, “Albahaca Morada” or combined with “clinical trial”, “clinical”, or “human”.

### 2.2. Inclusion Criteria

Studies were included if they reported on a human intervention study that involved ingestion of any form of tulsi or holy basil (*Ocimum sanctum* or* Ocimum tenuiflorum* or* Ocimum gratissimum*) and at least one clinical outcome.

### 2.3. Exclusion Criteria

Studies were excluded if they were reviews, were nonclinical studies, did not involve human subjects, did not report a biological outcome, only involved topical application or only used tulsi at part of a polyherbal formulation, and did not report on the use of tulsi as a single herbal intervention.

### 2.4. Study Selection

All the titles and abstracts were screened on the basis of the predetermined inclusion and exclusion criteria described above. The full text of each article was reviewed to assess suitability of the study with duplications removed. The search included clinical studies written in the English language and articles from inception until November 2016 in the above-mentioned electronic databases. The references of selected articles were manually searched to identify further relevant studies and, where appropriate, study authors were contacted to request further information.

### 2.5. Quality Assessment

In order to evaluate the quality of design and implementation of trials, information was collected on the study design, randomization, blinding, and description of participant dropouts and the Jadad scale was used to assess methodological quality [84].

### 2.6. Data Extraction

Eligible studies were reviewed with the following data extracted and tabulated: (1) first author name and year of publication; (2) design of the study; (3) Jadad score; (4) study participants (intervention and control groups); (5) extraction method; (6) duration of intervention; (7) tulsi dose and dose form (8) comparator; (9) outcome measure(s) including both primary and secondary, and (10) any adverse event(s).

## 3. Results

### 3.1. Study Description

**Figure 1:**
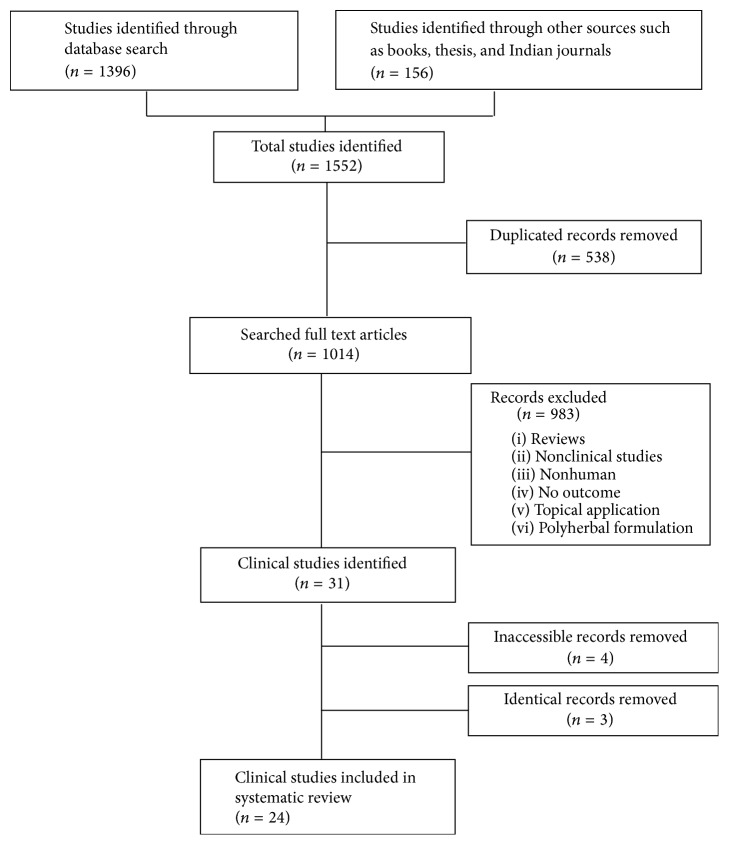
Flow chart of systematic search and study selection protocol.

After screening 1553 studies, a total of 31 articles on tulsi met the inclusion criteria. Four articles were excluded due to being inaccessible and one article reported two independent clinical studies, while one clinical trial was reported in three separate articles. This left a total of 24 independent clinical studies to be reviewed. A flow chart of the systematic search and study selection protocol is presented in Figure 1.

The reviewed studies involved a total of 1111 participants with ages ranging from 10 to 80 years old with eight clinical trials limiting participants to ≥40 years old [38, 61, 66, 68–70, 72, 75]. Only three clinical trials included 100 or more participants [65, 66, 82]. The study durations ranged from 2 to 13 weeks and tulsi dosage and frequency varied from 300 mg to 3000 mg given as 1–3 times per day as tulsi leaf aqueous extract; 300 mg–1000 mg once or twice per day as tulsi leaf ethanolic extract; 6 g to 14 g per day as the tulsi whole plant aqueous extract; and 10 g fresh tulsi leaf aqueous extract administered as once or four equal doses daily, and as tincture solution 30 drops a day were administered as three equal doses daily.

From the 24 studies identified only eight included a placebo [66, 68, 70, 75–77, 81, 82]. Five of the included trials adopted a two-arm parallel design [62, 63, 65, 67, 80], while four used a cross-over design with one being described in three different papers that reported on two different sets of outcomes [70, 75, 77].

Included studies were classified according to three major clinical domains: metabolic related disorders; immunity; and neurocognitive function (Tables 1, 2, and 3). Only two studies described the type of tulsi (Krishna) used while all other papers referred to tulsi as* Ocimum sanctum* [71, 72] not distinguishing between cultivars. Four studies reported on the use of tulsi alone and along with food, hypoglycemic drug, curry or Neem [63, 67, 69, 76]. Most studies looked at clinical populations with specific acute or chronic illnesses, such as viral infection, psychological stress, diabetes, or metabolic syndrome, with only three studies reporting on the effects of tulsi in healthy human participants [74, 76, 81].

### 3.2. Effectiveness and Safety Evaluation

The most common outcome measurements were related to blood glucose levels (8 studies), lipid profile (6 studies), blood pressure (6 studies), immune response (6 studies), and neurocognitive changes (4 studies). Other outcomes included mood (3 studies), fatigue (2 studies), uric acid levels (2 studies), diabetes secondary symptoms (1 study), and sleep (1 study). Fifteen of the 24 included studies reported no adverse events and eight studies did not describe or refer to any adverse events. Only one study that used tulsi leaf extract as 250 mg capsule taken before meals twice daily in 16 obese adults reported the occurrence of occasional nausea.

### 3.3. Quality Assessment

The studies were classified as either Randomized Clinical Trial Placebo Controlled (RCT-PC 6), Randomized Clinical Trials with no placebo (RCT 7), or Clinical Trials where no information on randomization or control was available (CT 6). Only three out of 24 studies, two of which examined neurocognitive effects [81, 82] and one reported on immunity as well as cardiovascular changes [74, 77], were rated as high quality with Jadad scores of 4-5 points, with the remaining studies varying in quality with Jadad scores ranging from 0 to 3 points. The score for each included study is presented in Tables 1–3.

### 3.4. Metabolic Disorders

Seventeen clinical trials reported on metabolic conditions with ten studies reporting on type 2 diabetes or metabolic syndrome with measures of blood glucose, lipids, and blood pressure, yet only one study reported on the clinical symptoms associated with type 2 diabetes such as polydipsia, polyphagia, polyuria, sweating, fatigue, burning feet, itching, and headache [69]. In addition one study reported on obesity [62] and two studies on uric acid changes in participants with gouty arthritis [38, 65].

Six of the identified trials on metabolic conditions were randomized clinical trials with placebo controls [66, 68, 70, 74, 75]. In addition, eight studies were of 2–5 weeks duration [38, 63, 64, 70, 73–75], three were of 6–8 weeks [61, 62, 68], and six were of 12-13 weeks [65–67, 69, 71, 72]. When the duration of the tulsi intervention was increased from 4-5 weeks [38, 70] to 12-13 weeks there was a more dramatic reduction in fasting blood glucose (FBG) and postprandial glucose (PPG) compared to controls [66, 67]. In particular, HbA1c (35.8%) significantly decreased when tulsi was added as adjunct therapy to hypoglycemic medication compared to drug medication alone [67].

The earliest clinical trial conducted in 1964 with 10 patients with type 2 diabetes reported that over a period of 12 weeks, a 14 g decoction of whole Krishna tulsi plant led to a gradual improvement in fasting blood glucose in 9 out of 10 patients [71]. Three decades later, the first randomized placebo-controlled clinical trial reported daily ingestion of 2.5 g of tulsi leaves led to significant improvements of FBG, PPG, and urine glucose in type 2 diabetes patients after 4 weeks [70]. In addition, Rai et al. reported that 4 weeks of supplementation with tulsi powder significantly lowered blood glucose and glycated proteins, reduced uric acid levels, and improved lipid profiles in participants with type 2 diabetes [38]. In comparable trials with longer durations, FBG and PPG improved by 1.2–2.2 and 1.5–6.0 folds, respectively, while HbA1c improved 1.5 and 3.2 fold after 12-13 weeks [66, 67]. Similarly, lipid profile was improved significantly in MetS and diabetes participants in three clinical trials [38, 66, 68] with a separate clinical trial reporting significant improvement in lipid profile in obese participants [62] and a further study reporting improved lipid levels in healthy subjects [74].

A further 12-week study of type 2 diabetes patients reported greater improvement in both blood glucose and HbAc1 levels when 300 mg of tulsi leaf extract was administered along with the antidiabetic drug glibenclamide, compared to drug treatment alone [67]. Similarly, a controlled trial of patients with diabetes found that consumption of 2 g of tulsi powdered leaves, either alone or combined with curry leaves, led to significant improvement in blood sugars after two weeks [63]. In a further 12-week randomized trial in diabetic patients, 2 g of tulsi leaf extract alone or combined with neem leaf extract produced marked reduction in diabetic symptoms with greatest effect noted for the combination [69].

Six trials reported on the effect of tulsi on individual features of metabolic syndrome [62, 72–75]. Two studies reported significant improvement of blood pressure in hypertensive participants given 30 mL of fresh tulsi leaf juice once daily or 30 mL twice a day for 10 and 12 days, respectively [75], with a further study reporting normalisation of blood pressure in hypotensive adult females [73]. Yet another study reported improvement in serum lipids with no difference in blood pressure in healthy adults administered 300 mg per day of tulsi leaf ethanolic extract for 4 weeks [74]. A further study reported improved lipid profiles in older adults (60–80 years) with psychosomatic symptoms after administration of 3 g of whole plant tulsi extract twice daily for 12 weeks [72]. A more recent study also reported improvement in lipid profiles, as well as BMI of obese participants administered 250 mg capsules of tulsi leaf extract twice daily for 8 weeks [62].

### 3.5. Immunomodulation and Inflammation

Enhanced immune response was reported in five clinical studies [76–80]. A small randomized double-blind, and placebo-controlled trial found increased immune response with increased Natural Killer (NK) and T-helper cells in healthy adult participants compared to placebo volunteers after 4 weeks of 300 mg or ethanolic tulsi leaf extract daily taken before food [77]. Another 2-week controlled randomized study in which young adult volunteers were provided with nutrition bars fortified with 1 g of ethanolic tulsi leaf extract found that compared to control participants, the intervention group had significantly improved VO_2_ max, less fatigue, reduced Creatine Kinase, and improved immune response to viral infection as indicated by reduced load of human herpesvirus 6 in saliva [76].

Two clinical trials studied the effect of daily administration of 10 g of an aqueous extract of fresh tulsi leaves in patients with acute viral infections, with a study on patients with acute viral encephalitis reporting increased survival after 4 weeks in the tulsi group compared to a group given dexamethasone and a study on viral hepatitis reporting symptomatic improvement after 2 weeks [79, 80]. A further study of asthmatic patients found that 500 mg of dried tulsi leaves taken three times daily improved vital capacity and provided relief of asthmatic symptoms within 3 days [78].

### 3.6. Neurocognitive Effect

The four studies that reported on neurocognitive effects all showed significant improvements in mood and/or cognitive function regardless of age, gender, formulation, dose, or quality of the study [72, 81–83]. Cognition function was assessed in a randomized, placebo-controlled, clinical trial that demonstrated an improvement in cognitive flexibility, short-term memory, and attention in 40 healthy young adults (17–30 years) following treatment with 300 mg daily tulsi for 4 weeks [81]. However, the cognitive effects of tulsi were only significant after the first two weeks compared to the placebo, with no significant difference found in stress levels. This is in contrast to three clinical studies that reported significant reduction in anxiety and stress levels with higher doses of tulsi given over a longer time period [72, 82, 83]. The positive effect of tulsi on mood was demonstrated in three studies, with two studies reporting reductions of 31.6%–39% in overall stress-related symptoms in patients with psychosomatic problems compared to a control group [82, 83].

## 4. Discussion

Despite a long history of traditional use and widespread availability, relatively few human intervention studies have been conducted on the effectiveness of tulsi for clinical conditions and this is the first comprehensive literature review of published human research on the ingestion of tulsi as a single herbal intervention. The studies identified in this review could be classified according to three main clinical domains including metabolic disorders (15 studies), neurocognitive or mood conditions (4 studies), and immunity and infections (5 studies), which are all extremely relevant to the growing world-wide epidemic of lifestyle-related chronic disease. The finding that the reviewed studies reported favourable clinical effects across these domains suggests that tulsi may indeed be an effective adaptogen that has a role in helping to address the psychological, physiological, immunological, and metabolic stresses of modern living.

It is interesting that tulsi has important clinical effects across diverse therapeutic domains, all of which may have inflammation as an underlying factor. The anti-inflammatory effects of tulsi have been previously documented in many in vitro and in vivo studies [43, 85–88], and it is likely that tulsi has multiple bioactive secondary metabolites that act alone or synergistically to inhibit inflammatory pathways. There is also evidence to suggest that tulsi may be useful as an adjunct to pharmacotherapy and nutrition in the treatment of metabolic disorders thereby reducing the need for high doses of drugs, which may have adverse effects. The clinical effects demonstrated in the reviewed studies suggest tulsi may have an important role in addressing other inflammatory disorders and that the Ayurvedic tradition of consuming tulsi on a daily basis may be an effective lifestyle measure to address many modern chronic diseases.

The most commonly used part of the tulsi plant is the leaf (dried or fresh), which is known to contain several bioactive compounds including eugenol, ursolic acid, *β*-caryophyllene, linalool, and 1,8-cineole [89–91]. Eugenol has been found to be the major bioactive metabolite common to all three tulsi varieties with varying amounts in each cultivar [92, 93] and it has recently been suggested to act via dual cellular mechanisms to lower blood glucose levels. These include competitively preventing the binding of glucose to serum albumin and inhibiting the conversion of complex carbohydrate to glucose [93]. However, while eugenol has been shown to be bioactive, the phytochemical composition of tulsi is very complex and varies depending on different conditions [94–97] and there are many other potential active secondary metabolites such as other phenylpropanoids (methyl eugenol, rosmarinic acid), monoterpenes (ocimene), and sesquiterpenes (germacrene) that could alone or synergistically produce therapeutic benefits [98].

All reviewed studies reported favourable clinical effects with minimal or no side effects irrespective of dose, formulation, or the age or gender of participants, with only one clinical trial reporting transient mild nausea [62]. As the longest study was only 13 weeks, the failure to report any adverse effects does not preclude the presence of any long term side effects; however, the long traditional history of regular tulsi use suggests any serious long term effects are unlikely and that daily ingestion of tulsi is safe. Furthermore, the results of this review are consistent with previous evidence for the clinical efficacy and safety of tulsi, which includes multiple in vitro and in vivo studies and many human clinical trials in addition to traditional use.

### 4.1. Limitations and Scope

This review, which comprehensively reviewed all human clinical trials published in English language on ingestion of tulsi as a single herb, has many limitations. While the review included 24 studies and minimized bias by using a systematic and independent search strategy without limiting publication year or study design, we cannot be certain that all studies were located; this is especially due to the fact that almost all studies were conducted in India and published in local journals, some of which are very difficult to access or search. There may also be unpublished studies that report negative outcomes [99]. Furthermore, while the reviewed studies were consistent in reporting positive effects of tulsi in humans, only 7 out of 24 can be considered high quality studies with all but three failing to include a double-blind strategy. Tulsi's therapeutic effects may have therefore been overestimated and while the efficacy of tulsi was reported across a wide range of formulations and doses, many studies also failed to provide details of the cultivar, dosage form, or specific dosage or quality control measures of the tulsi used.

This review suggests that tulsi is an example of the Ayurvedic holistic lifestyle approach to health and appears to provide a vast array of health benefits that offers solutions to many modern day health problems. While the reviewed studies could be classified into three major therapeutic domains, there is insufficient evidence for any specific tulsi formulation to assist in any one condition. More rigorous studies with larger sample sizes and longer durations and standardised formulations are therefore needed before specific recommendations can be made for the treatment of any specific disease. This review further highlights the need to investigate and determine unique signature compounds specific to each of the three tulsi varieties, to not only identify the bioactive metabolites that may synergistically interact, but also shed light on the underlying mechanism of action on metabolic and inflammatory pathways.

## 5. Conclusion

Despite the lack of large-scale or long term clinical trials on the effect of tulsi in humans, the findings from 24 human studies published to date suggest that the tulsi is a safe herbal intervention that may assist in normalising glucose, blood pressure and lipid profiles, and dealing with psychological and immunological stress. Furthermore, these studies indicate the daily addition of tulsi to the diet and/or as adjunct to drug therapy can potentially assist in prevention or reduction of various health conditions and warrants further clinical evaluation.

## Figures and Tables

**Table 1:** Effect of tulsi on metabolic related-disorders in human clinical trials.

Clinical domain	Authors (year)	Study design	Jadad score	Participants^*∗∗*^ (age range)	Tulsi extract	Intervention		Comparator	Outcome measure(s)	Adverse events (s)
						Duration^*∗*^	Dosage			
Metabolic disorders	Gandhi et al. (2016) [61]	Randomized controlled clinical trial	**1**	40 male adults T2DM (45–55 years)	Tulsi leaves caps	6.5 weeks	3 g/day before meal	Not disclosed	Significant↓ postprandial glucose & fasting blood glucose	Not reported
	Satapathy et al. (2016) [62]	Randomized parallel group clinical trial	**3**	30 adults Obesity (17–30 years)	Tulasi^1^ leaves caps	8 weeks	250 mg/day 2x daily before meal	Parallel group no intervention	Improved BMI, lipid profile (except TC), TG & IR	Mild nausea
	Venkatesan and Sengupta (2015) [63]	Clinical trial controlled parallel group	**0**	30 adults T2DM	Tulsi powder leaves	2 weeks	2 g/day	Curry leaves tulsi + curry	Significant↓ post-prandial glucose & fasting blood	Not reported
	Srinivasa Prasadacharyulu (2014) [64]	Clinical study case report	**0**	3 adults T2DM	Fresh tulsi leaves	5 weeks	fresh leaves^3^ 3x daily	None	Considerable decrease in blood glucose reaching near normal levels	None
	Ahmad et al. (2013) [65]	Randomized single-blind parallel group	**2**	200 adults Gouty Arthritis	Tincture^2^ from tulsi	12 weeks	10 drops 3 times/day	Tincture wild rosemary 10 drops × 3/day	Significant reduction in serum uric acid	Not reported
	Devra et al. (2012) [66]	Randomized, placebo-controlled clinical trial	**1**	100 adults MetS (≥40 years)	Aqueous tulsi Leaves	12 weeks	5 mL/×2 day before meals	Not disclosed	Improved lipid profile, fasting blood glucose, and BP	Not reported
	Somasundaram et al. (2012) [67]	Randomized, controlled parallel clinical Trial	**1**	60 adults T2DM (30–65 years)	Tulsi leaves + glibenclamide drug	13 weeks	300 mg/day tulsi + 5 mg Glibenclamide	5 mg/day glibenclamide	Significant ↓fasting blood & postprandial glucose reduced HBA1c	None
	Dineshkumar et al. (2010) [68]	Randomized placebo-controlled clinical trial	**3**	40 adults T2DM (45–55 years)	Aqueous tulsi leaves	8 weeks	500 g/day	Water	Significant improvement in lipid profile	None
	Kochhar et al. (2009) [69]	Randomized, clinical trial	**1**	90 male adults T2DM/MetS (40–60 years)	Powder tulsi leaves	12 weeks	2 g/day	Neem neem + tulsi	Improved T2DM symptoms: ↓polydipsia, ↓polyphagia, & BP	Not reported
	Rai et al. (1997) [38]	Clinical trial controlled group	**1**	27 adults T2DM/MeS (45–65 years)	Tulsi powder leaves	4 weeks	1 g/day in morning before meal	None	Improved lipid profile, blood glucose, glycated proteins (HbA1c) & UA	Not reported
	Agrawal et al. (1996) [70]	Randomized, single-blind, Placebo-controlled cross-over	**1**	40 adults T2DM (41–65 years)	Tulsi powder leaves	5 weeks (+5-day wash out)	2.5 g/day in morning before meal	Spinach powder leaves 2.5 g/day	Significant ↓fasting blood glucose, post-prandial glucose and urine glucose	None
	Luthy (1964) [71]	Clinical trial	**1**	10 adults T2DM	Whole plant decoction	12 weeks	14 g/day	None	Reduced blood glucose in 9 T2DM adults	None
	Verma et al. (2012) [72]	Clinical trial	**0**	5 adults psychosomatic (60–80 years)	Powder whole plant tulsi	12 weeks	3 g × 2/day	None	Significant improvement in lipid profile	None
	Bhargava et al., (2013) [73]	Clinical study open label	**1**	50 Female adults hypotensive (18–30 years)	Fresh juice 15 tulsi leaves	4 weeks	Juice × 2/day	None	Significant changes, in Blood pressure	Not reported
	Mondal et al. (2012) [74]	Randomized, double-blind, placebo-controlled cross-over leaves	**5**	22 healthy adults (22–37 years)	Ethanolic tulsi	4 weeks (+3-week wash-out)	300 mg/day before food	300 mg/day sucrose	Reduction in lipid profile, in 6 participants	None
	Sarvaiya (1986) [75]	Randomized placebo controlled cross-over	**1**	20 adults, hypertension (45–64 years)	Fresh Juice 75% tulsi	10 days (+5-day wash-out)	30 mL/day	Green colored water 30 mL/day before meal	Significant ↓blood pressure	None
	Sarvaiya (1986) [75]	Randomized placebo-controlled	**1**	16 adults, hypertension (45–64 years)	Fresh Juice 75% tulsi	12 days	30 mL 2 times a day before meals	Green-colored water 30 mL × 2/day	Significant ↓blood pressure (lowered by 25%)	None

BMI = body mass index measured by weight (kg)/height (m^2^); BP = blood pressure; HbA1C = glycosylated haemoglobin; IR = insulin resistance; MetS = metabolic syndrome; T2DM = type 2 diabetes mellitus; TC = total cholesterol, TG = triglycerides; UA = uric acid.

^*∗*^ *Intervention duration* is the time the intervention was administered excluding any washout periods.

^*∗∗*^ *Participants* who completed the study are listed excluding any drop-outs.

^1^ *Tulasi Tablets* are product of Himalaya Herbal Healthcare Pharmaceutical Company in India.

^**2**^Tincture is a product of BM private limited.

^3^The quantity of tulsi fresh leaves was not specified; “handful” of leaves was given to each patient.

**Table 2:** Effect of tulsi on immune system and viral infections in human clinical trials.

Clinical domain	Authors (year)	Study design	Jadad score	Participants^*∗∗*^ (age range)	Tulsi extract	Intervention		Comparator	Outcome measure(s)	Adverse events (s)
						Duration^*∗*^	Dosage			
Immunomodulation	Venu Prasad (2014) [76]	Randomized, placebo-controlled clinical trial	**3**	30 healthy adults (18–30 years)	Ethanolic tulsi leaves in Bar^‡^	2 weeks	1 bar × 2/day (1000 mg tulsi)	Not described “control bar”	↑physical performance ↓fatigue and CK levels less increase in lactic acid	None
	Mondal^†^ et al. (2011) [77]	Randomized, double-blind, placebo-controlled cross-over	**5**	22 healthy adults (22–37 years)	Ethanolic tulsi leaves	4 weeks (+3 weeks wash out)	300 mg/day	Cellulose 300 mg/day	Increased cytokine level, interferon-*ϒ*, & interleukin-4	None
	Sharma (1983) [78]	Open clinical trial	**1**	20 adults, asthma	Aqueous tulsi leaves tablets	1 week	500 mg × 3/day	None	Relief within 3 days, improved vital capacity	None

Viral infections	Rajalakshmi et al. (1986) [79]	Clinical trial	**0**	20 cases, viral hepatitis (10–60 years)	Aqueous extract fresh tulsi leaves	2 weeks for mild cases 3 weeks for Severe cases	10 g daily	None	Symptoms all improved within 2 weeks	None
	Das et al. (1983) [80]	Randomized clinical trial parallel-controlled	**1**	14 adults, viral encephalitis	Aqueous extract fresh tulsi leaves	4 weeks	2.5 g 4 times/day	12 mg/day dexamethasone treated group	Increased survival rate compared to steroid	Not reported

CK = creatine kinase; TPE = tropical pulmonary eosinophilia.

^*∗*^ *Intervention duration* included wash-out periods where applicable until study was completed.

^*∗∗*^ *Participants* include both control and intervention groups completing the study and excluded any drop-outs.

^†^Same results as previously published (Mondal et al., 2010).

^‡^Tulsi enriched bar: each 25 g bar contained oats, resin, peanuts, skimmed milk powder, sugar, and honey and 0.5% ethanolic tulsi.

**Table 3:** Therapeutic effects of tulsi on cognitive function, mood, and stress in human clinical trials.

Clinical domain	Authors (year)	Study design	Jadad score	Participants^*∗∗*^ (age range)	Tulsi extract	Intervention		Comparator	Outcome measure(s)	Adverse events (s)
						Duration^*∗*^	Dosage			
Neurocognition	Sampath et al. (2015) [81]	Randomized, double-blind, placebo controlled clinical trial	**5**	40 healthy adults (18–30 years)	Ethanolic tulsi leaves capsules	4 weeks	300 mg/day before meals	Cellulose capsules	Cognitive flexibility, attention, Improved working memory only after day 15	None
	Saxena et al. (2012) [82]	Randomized, double-blind, placebo-controlled	**4**	150 adults, stress (18–65 years)	OCIBEST^†^ whole plant capsules	6 weeks	400 mg 3 times/day after meals	Cellulose capsules	Reduction in stress related symptoms: fatigue, sleep and sexual problems	None
	Verma^‡^ et al. (2012) [72]	Clinical trial	**0**	24 adults, psychosomatic (60–80 years)	powder whole plant tulsi	12 weeks	3 g × 2/day	None	Reduced anxiety significantly lowered biological age score	None
	Bhattacharyya et al. (2008) [83]	Clinical trial	**1**	35 adults with GAD (18–60 years)	Ethanolic tulsi leaves capsules	8 weeks	500 mg 2x daily after meals	None	Self-reported questionnaire, ↓anxiety, stress, & depression	None

GAD = generalized anxiety disorder.

^*∗*^ *Intervention duration* is the time the intervention was administered excluding any washout periods.

^*∗∗*^ *Participants* include both control and intervention groups completing the study and excluded any drop-outs.

^†^ *OCIBEST* is product of natural remedies and contains tulsi whole plant.

^‡^Authors also reported findings from glucose and lipid profile for 5 of the participants and found lipid profile significantly improved.
